# Evaluation of Polymyxin B as a Novel Vaccine Adjuvant and Its Immunological Comparison with FDA-Approved Adjuvants

**DOI:** 10.3390/vaccines13121232

**Published:** 2025-12-09

**Authors:** Mahek Gulani, Yash Harsoda, Tanisha Arte, Martin J. D’Souza, Priyal Bagwe, Emmanuel Adediran, Nigel D’Souza, Dedeepya Pasupuleti

**Affiliations:** 1 Vaccine Nanotechnology Laboratory, College of Pharmacy, Center for Drug Delivery Research, Mercer University, Atlanta, GA 30341, USA; mahekanil.gulani@live.mercer.edu (M.G.); yashkumar.pankajbhai.harsoda@live.mercer.edu (Y.H.); tanisha.manoj.arte@live.mercer.edu (T.A.); dsouza_mj@mercer.edu (M.J.D.); priyal.bagwe@live.mercer.edu (P.B.); 2 Department of Pharmaceutical Sciences, Larkin College of Pharmacy, 18301 North Miami Ave, Miami, FL 33169, USA; eadediran@larkin.edu; 3 Memorial Health University Medical Center, Savannah, GA 31404, USA; nigel.dsouza@hcahealthcare.com

**Keywords:** Polymyxin, Polymyxin B, adjuvant, mucosal adjuvant, vaccines, microparticles, repurposing drugs, vaccine adjuvant, flow cytometry, MHC I MHC II

## Abstract

Background: Adjuvants enhance the immune response to antigens incorporated in vaccine formulations. Given that the majority of infectious agents enter the body through mucosal surfaces, efficacious vaccines must generate protective immunity at these sites, which serve as the first line of defense. There is a need for mucosal adjuvants; hence, we explored the potential of repurposing existing drugs with established safety profiles in humans. Polymyxins are a class of clinically used antibiotics. They are cationic peptides and mast cell activators, which are a novel class of vaccination adjuvants. The goal was to assess the adjuvant properties of Polymyxin B microparticles in combination with vaccine candidates previously developed in our laboratory, such as microparticulate gonorrhea, influenza, measles, Zika, and canine coronavirus vaccines, and to compare their performance with FDA-licensed adjuvants, such as MF59 and Alum. Methods: Polymyxin microparticles were formulated using a double emulsion method, and the toxicity profile and autophagosome generation of Polymyxin B microparticles were assessed. The immunogenic potential of Polymyxin B in these vaccines was studied using multiple parameters such as nitric oxide release using Griess assay and immune profiling using flow cytometry for markers such as MHC I, MHC II, CD40, and CD80. Results: Polymyxin B microparticles were found to be non-cytotoxic to dendritic cells up to 500 μg/mL. Polymyxin B promoted autophagosome formation and nitric oxide release, and showed the upregulation of MHC I, MHC II, CD80, and CD40 pathways. Conclusions: The adjuvant activity of Polymyxin B across various vaccine platforms is significantly comparable to FDA-approved adjuvants, which is a critical requirement for generating T cell responses such as helper T cell and cytotoxic CD8+ T cell responses.

## 1. Introduction

Adjuvants are substances that improve vaccine antigen immunogenicity and stability while increasing the intensity of immune responses to the antigen. The Latin term adjuvare, which means to assist, is where the word adjuvant first derived. Adjuvants increase antibody avidity and specificity while lowering the dose of vaccination antigens and the frequency of doses required for protective immunization. Adjuvants are often divided into two classes based on how they work: vaccine delivery methods and immunostimulatory adjuvants. The immune response is directly boosted by adjuvants with immunostimulatory properties, including immunostimulatory complexes, which are a self-assembled non-covalent mixture of phosphatidylcholine, mineral salts, microorganism-derived chemicals, and saponins from cholesterol, etc. However, because of their special delivery characteristics such as particle size, vaccine delivery technologies like micro/nanoparticles and liposomes help to improve the response [1,2,3]. Adjuvants are thought to work through a number of processes to increase vaccination immunogenicity: they have the ability to produce a depot effect, which permits the gradual release of antigens from the administration site; they promote the release of cytokines and chemokines; and they induce local inflammation at the application site, which aids in the recruitment of immune cells as well as the uptake and presentation of antigens to antigen-presenting cells (APCs) [4,5,6]. APC antigen uptake is improved, and adjuvants boost phagocytosis and pinocytosis. Adjuvants also activate inflammasomes and increase the expression of costimulatory molecules and MHC class II, which start the maturation and activation of APCs. Adjuvants boost the activation and proliferation of cytotoxic T cells, lengthen and speed up the immune response, and enhance the immunological response in elderly, immunocompromised, and neonatal patients [7,8,9].

Most infectious agents enter through mucosal surfaces, so vaccines aim to elicit immunity at these sites, including secretory antibodies that function as the initial defense [10,11]. For pathogens transmitted across mucosal barriers like COVID-19 and influenza, mucosal immunization is critical, as it promotes pathogen-specific protection mainly through the generation of secretory IgA [12]. The coadministration of potent mucosal adjuvants can improve protective immune responses at mucosal surfaces. Enterotoxins, for example, heat-labile toxin (LT), are potent mucosal adjuvants that boost systemic and mucosal immune responses to co-administered protein antigens. But when a commercial intranasal vaccination that utilized LT as an adjuvant was taken off the market because of a potential link to side effects like headaches, rhinorrhea, and, most importantly, facial palsy, safety concerns were raised. There are currently no clinically available mucosal adjuvants [13,14]. We concentrated on currently available medications that have undergone human testing and are therefore reasonably safe in order to create safer mucosal adjuvants [15].

Polymyxins are well-known antibiotics used in therapeutic settings. Since the 1950s, Polymyxin B has been utilized in clinical settings. Polymyxins are used to treat illnesses brought on by bacteria that are resistant to many drugs because they are highly bactericidal against Gram-negative organisms [16,17,18].

It has long been known that polymyxins, which are cationic peptides, are mast cell activators since they have been demonstrated to trigger the release of histamine and other signals from isolated mast cells as well as their degranulation. Mast cells have gained attention lately as a novel participant in vaccination-induced antigen-specific adaptive immune responses. Their activation not only triggers the innate immune response, but also causes immune cells to migrate into draining lymph nodes, which, in turn, triggers the adaptive immune response. These cells interact with diverse immune cells comprising dendritic cells, macrophages, lymphocytes, and Langerhans cells. On the other hand, cationic peptides are potent compounds that have a role in numerous areas of innate immunity. For instance, it has been demonstrated that certain cationic peptides promote the chemoattraction of neutrophils and monocytes, while others enhance macrophage nonopsonic phagocytosis. These results have led to the consideration of cationic peptides and mast cell activators as a novel class of vaccination adjuvants [14,19].

Given the significant costs and slow advancement of new drug discovery and development, repurposing ‘old’ drugs for new therapeutic indications is emerging as an appealing strategy. This strategy leverages de-risked compounds—agents with already established safety profiles, pharmacokinetics, and manufacturing processes—thereby reducing both the financial burden and the time required for development. Multiple data-driven strategies and experimental methods have been proposed to identify repurposable drug candidates. Together, these strategies provide a powerful framework for expanding the therapeutic utility of existing drugs while accelerating the pipeline to clinical use [20,21,22].

There are limited studies on repurposing Polymyxin B as a vaccine adjuvant. The studies conducted to date include research by Yoshino et al. [14], who demonstrated that Polymyxin B considerably enhanced the generation of IgA in the mucosal secretions of vaccinated mice, demonstrating the mucosal adjuvanticity of Polymyxin B for influenza HA and SARS-CoV-2 S proteins in comparison to the antigen only. There were no negative effects on the olfactory bulb or nasal mucosa. These results demonstrate that Polymyxin B has the potential to be a safe option for intranasal immunization in order to produce mucosal IgA antibodies for the prevention of mucosally transmitted illnesses. Yoshino et al. [13] also demonstrated that Polymyxin B’s mucosal adjuvanticity is influenced by both the hydrophilic cationic cyclic peptide and the hydrophobic carbon chain. However, a notable gap in the literature is the lack of direct comparisons between Polymyxin B and clinically approved adjuvants, namely, Alum and MF59, which have long been established as safe and effective components of licensed human vaccines.

The objective of this study is to investigate the potential of Polymyxin B as a novel vaccine adjuvant. We investigate its immunomodulatory effects across a range of bacterial and viral vaccine candidates, including gonorrhea, influenza, measles, COVID-19, and Zika. Specifically, we compare the immune responses elicited by Polymyxin B-adjuvanted antigens to those generated with Alum- or MF59-adjuvanted antigens. In addition, we assess the impact of Polymyxin B when used in combination with these adjuvants.

## 2. Materials and Methods

### 2.1. Materials

Aldrich Sigma (Burlington, MA, USA) was the supplier for the dichloromethane, polyvinyl alcohol, Polymyxin B, and trehalose needed to create the nanoparticles using the double emulsion process. Evonik Industries provided the 75:25 poly(lactic-co-glycolic) acid. Fisher Scientific (Waltham, MA, USA) was the supplier of bovine serum albumin (BSA). Murine dendritic cells (DC 2.4) were offered as a gift by Dr. Kenneth L. Rock (Dana-Farber Cancer Institute Inc., Boston, MA, USA). The ATCC also provided the cell culture supplies, which included penicillin and streptomycin, Dulbecco’s Modified Eagle’s Medium, and fetal bovine serum. We bought MF59^®^ and Alum from InvivoGen in California. BEI Resources provided the canine coronavirus and influenza H1N1 antigens used in this study. The Serum Institute of India, located in India, is where the measles vaccine was bought. The strain CDC-F62 of *Neisseria gonorrhoeae* was supplied by Dr. William Shafer in Georgia, USA. The Centers for Disease Control and Prevention provided the Zika virus strain PRVABC59. Thermo Fisher Scientific (Rockford, IL, USA) provided the sodium nitrite, sulfanilamide, and N-1-naphthyl ethylenediamine dihydrochloride. From Sigma Aldrich (St. Louis, MO, USA), 3-(4,5-dimethylthiazol-2-yl)-2,5-diphenyl tetrazolium bromide was acquired. For flow cytometric analysis, markers including MHC I, MHC II, CD40, and CD80 were acquired from eBioscience laboratories (San Diego, CA, USA).

### 2.2. Methods

#### 2.2.1. Formulation of Bacterial Whole-Cell Inactivated *N. gonorrhoeae* Vaccine Microparticles and Viral Measles Vaccine

The Buchi Mini Spray Dryer was used to make the biodegradable microparticles (MPs) and the formalin-inactivated whole-cell *N. gonorrhoeae* antigen. Vaccine microparticles at 10% antigen loading were formulated by dissolving 180 mg of BSA in 10 mL of deionized water. BSA serves as the biodegradable, albumin-based polymer matrix that mimics antigen conjugation to a protein carrier. Because APCs express albumin-binding receptors, the BSA matrix facilitates microparticle uptake and enhances antigen internalization, thereby promoting a stronger cellular immune response. After dissolving the BSA, 400 µL of glutaraldehyde was added for every 2 gm of BSA, and the mixture was stirred at 300 rpm for the entire night at room temperature in a dark location. Then, 20 mg of sodium bisulfate was used to neutralize the extra glutaraldehyde. At this stage, the overnight-prepared pre-crosslinked BSA was combined with the *N. gonorrhoeae* antigen. Following this, 10 mL of deionized water was used to dissolve 100 mg of this produced mixture. A 0.5 mm nozzle was used to spray-dry the mixture (nozzle temperature: −5 °C). The *N. gonorrhoeae* vaccine microparticles were obtained at an incoming temperature of 120 °C and flow rate of 20 mL/h with the aspirator operating at 100%. The preparation of measles vaccine microparticles (5% *w*/*w* of the measles Edmonston–Zagreb strain antigen) were spray-dried with the same parameters.

#### 2.2.2. Formulation of Adjuvants and Viral Vaccine Microparticles for the H1N1 (Influenza A Strain) Virus, Canine Coronavirus (iCCoV), and Zika Virus

Using a previously developed double emulsion solvent evaporation approach, separate microparticles (MPs) including the deactivated Influenza A H1N1 antigen (BEI resources), iCCoV antigen (BEI resources), Zika antigen, Alum, MF59, and Polymyxin B adjuvants were formulated [23]. In short, the initial emulsion consisted of dichloromethane (DCM) solution, poly(lactic-co-glycolic) acid (PLGA), and the antigen/adjuvant (2 percent loading) created by high-speed homogenization. This emulsion was added to polyvinyl alcohol solution in water and homogenized again, creating a double emulsion. The double emulsion was continually stirred to evaporate any leftover DCM, after which the double emulsion was ultracentrifuged and the pellet was reconstituted in trehalose solution. A lyophilizer was then used to freeze-dry the final microparticulate vaccine formulation, producing a dry powder.

#### 2.2.3. Physicochemical and Morphological Characterization of Polymyxin B Microparticles

The weight of the microparticles was recorded both before and after the lyophilization procedure. The practical yield is calculated by comparing the weight of the lyophilized microparticles to the total weight of all components used in the formulation [24].

Scanning electron microscopy was then used to visualize the formed MPs for shape and morphology. The MPs were attached to the stubs using 12 mm double-sided carbon tabs. The PhenomTM benchtop scanning electron microscope was then used to view the MPs.

Using a Zetasizer (Malvern Instruments Inc., Westborough, MA, USA), the nanoparticle size, polydispersity index, and zeta potential were measured three times simultaneously. Here, 1 mL of deionized water was used to dilute 50 μL of the sample (1 mg/1 mL). Room temperature was used for the measurements [25].

Polymyxin B MPs were analyzed by Fourier-transform infrared (FTIR) spectroscopy (Shimadzu IRAffinity-1S, Tampa, FL, USA). Free Polymyxin B, blank MPs, and Polymyxin B MPs were measured. In summary, 1 mg of the sample was kept for examination on a ZnSe crystal puck. Following three runs of this sample, the spectra of each were measured. The percentage transmittance was plotted against the wavenumber in order to identify changes in critical functional groups that would indicate significant interactions between the excipients and Polymyxin B in the microparticles [26].

#### 2.2.4. In Vitro Release Profile of Polymyxin Microparticles

To determine the amount of Polymyxin B that was released from the PLGA polymer, a release study of Polymyxin B was carried out [27]. A standard curve of Polymyxin B in PBS was plotted for calculation. To perform the release study, 5 mg of Polymyxin B MP was put to a beaker with 20 mL of PBS. After that, the beaker was placed on a plate shaker set to 60 rpm and 37 °C. Samples were taken and replaced with fresh PBS at predetermined intervals (0.25 to 60 h). The sample was centrifuged at 1500 rpm for 8 min. After eight minutes, the collected supernatant was measured using a UV spectrophotometer set to 210 nm (NANODROP 2000c, Thermo Fisher).

#### 2.2.5. Assessment of the Cytotoxicity of Polymyxin B Microparticles

The MTT assay [28] was used to investigate whether Polymyxin B microparticles are toxic to cells. Here, 10,000 dendritic cells were introduced into each well of a 96-well plate on the first day. The following day, the cells were exposed to a range of treatments at various Polymyxin B microparticle concentrations, from 62.5 to 500 µg/mL (*n* = 3 per group), including cells alone serving as a negative control and dimethyl sulfoxide (DMSO) serving as a positive control. The cells were then incubated at 37 °C and exposed for a whole day. The third day involved washing the wells, treating them with 10 µL of 0.5% *w*/*v* MTT reagent, and adding media to increase the level to 200 µL. The plate was then incubated for four hours to enable the production of the purple precipitate by metabolically living cells. After adding 100 µL of DMSO to solubilize the precipitate, the plate was wrapped in foil and left on a shaker for 20 min. The measurement was taken at 570 nm, and the cell viability was calculated.

#### 2.2.6. Qualitative and Quantitative Evaluation of Autophagosomes

It has previously been observed and documented how autophagy controls dendritic cell processes such maturation, cytokine generation, antigen presentation, and subsequent T cell activation. Antigen presentation through MHC class II molecules and antigen cross presentation through the MHC class I pathway are associated with autophagy [29,30,31]. Here, a CYTO-ID^®^ autophagy detection kit was used as recommended by the manufacturer to evaluate the production of autophagosomes in dendritic cells after stimulation with various treatment groups. Flow cytometry was used for the quantitative study, while fluorescent live cell imaging was used to provide the qualitative images. For qualitative imaging, dendritic cells were seeded at a density of 5 × 10^4^ cells in a 24-well plate on the first day. Cells only, Polymyxin B suspension, and Polymyxin B MP were the treatments given to the cells after a 24 h period. Each group’s dosage matches the dose listed in Table 1 for the respective group. The treated cells were incubated overnight, and on the last day of the experiment, the cells were stained with CYTO-ID^®^ dye and Hoechst Nuclear stain for 30 min at room temperature after being rinsed with PBS. Following the incubation time, the non-bound dye was cleaned, and the cells were examined using a fluorescence microscope’s DAPI and FITC filters. Hoechst nuclear stain was used to stain the cell nucleus, which appeared blue, and CYTO-ID^®^ dye was used to stain the autophagosomes, which appeared green.

The same procedure was used to measure the quantitative expression of autophagosomes in dendritic cells, with the exception that the cells were solely labeled with the CYTO-ID^®^ dye. The groups that were used were cells only, Polymyxin B solution, Polymyxin B MP, Alum MP, H1N1 MP, H1N1 + Alum MP, and H1N1 + Polymyxin B MP. Following gentle trypsinization, the cells were subjected to flow cytometry (BD Accuri C6 Plus flow cytometerBecton, Dickinson and Company, San Diego, CA, USA) to determine the percentage of cells expressing autophagosomes.

#### 2.2.7. In Vitro Immunogenicity Evaluation of Adjuvant Effect Using Griess Assay for Nitrite Detection

Griess’ assay, a standard evaluation of the stimulation of innate immunity, was used to compare the immunostimulatory capability of Polymyxin B MP to FDA-licensed adjuvants. Antigen-presenting cells emit nitric oxide, which may be measured by its nitrite—an oxidation product produced after MPs are exposed to these cells [32,33]. By measuring the nitric oxide released from dendritic cells after exposure to Polymyxin B microparticles in combination with iCoV, H1N1 influenza, Zika, measles, and gonorrhea vaccines, the adjuvant impact of the Polymyxin B MPs was examined. In short [33], DC 2.4 were cultured for 24 h in a 96-well plate. These cells were then subjected for 24 h to the microparticle groups in Table 1. The supernatants were moved to a different plate after 24 h, and the Griess reagent was added. A BioTek Synergy plate reader was utilized to record absorbance at 540 nm after the plate had been incubated in the dark for 10 min. A standard curve made with a sodium nitrite solution was applied to determine the content of nitrite.

#### 2.2.8. Evaluation of Antigen-Presenting and Co-Stimulatory Molecule Expression via Flow Cytometry

Flow cytometric analysis was performed to assess the upregulation of antigen-presenting and co-stimulatory molecule expression induced by Polymyxin B MPs. Dendritic cells were cultured on a 48-well plate and cultured for 24 h at 37 °C to adhere the cells. Next, as indicated in Table 1, these adherent cells were stimulated with (n = 3 per group) microparticles in each well, and they were incubated for 24 h at 37 °C. Post a third-day wash to eliminate extracellular debris, the exposed cells were stained with allophycocyanin (APC)-labeled CD40 and CD80 markers and FITC-labeled MHC I and MHC II markers, respectively. After washing the labeled cells, the BD Accuri C6 flow cytometer was used to measure the fluorescence intensity.

#### 2.2.9. Statistical Analysis

GraphPad Prism 10 was employed for the statistical analysis of all experimental data. The mean ± standard error of the mean (SEM) was used to express the following results. Three replicates of each experiment were carried out. Tukey’s post hoc test was used after a one-way ANOVA when comparing several groups. The *p*-values were considered significant in the graphs created with GraphPad software: *p* > 0.05 (ns, nonsignificant), *p* ≤ 0.05 (*), *p* ≤ 0.01 (**), *p* ≤ 0.001 (***), and *p* ≤ 0.0001 (****). A *p*-value of less than 0.05 was deemed statistically significant in every experiment.

## 3. Results

### 3.1. Physiochemical and Morphological Characterization of Polymyxin B Microparticles

**Figure 1:**
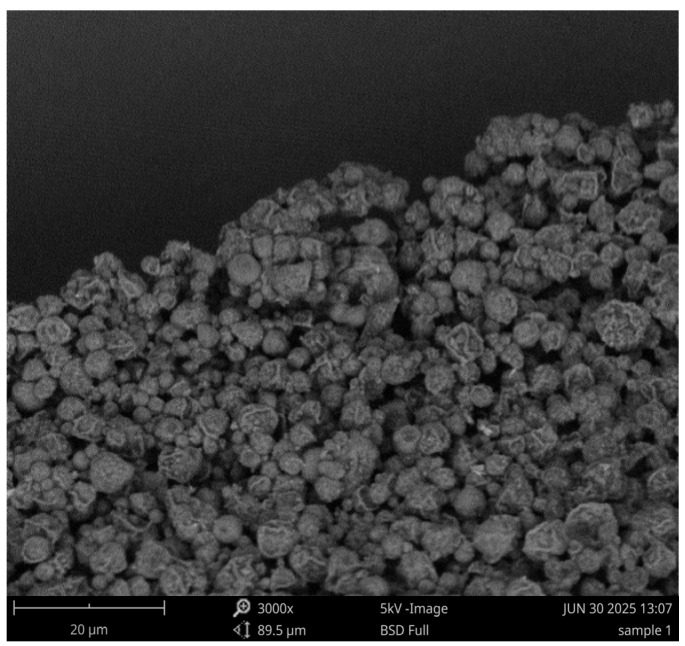
Scanning electron microscopy image of Polymyxin B microparticles prepared using the double emulsion method. The particles exhibit a spherical morphology with an average diameter of 2017 nm, PDI of 0.296, zeta potential of −22.6 mV, and a percent recovery yield of 78% after lyophilization.

The particle size of the Polymyxin B microparticles was found to be 2017 nm, and the formulation showed a recovery yield of 78% with a polydispersity index of 0.296. The zeta potential was recorded at –22.6 mV (Table 2). The surface morphology of Polymyxin B microparticles was examined using scanning electron microscopy (Figure 1), which revealed spherical particles with a smooth structure and no visible deformities.

**Figure 2:**
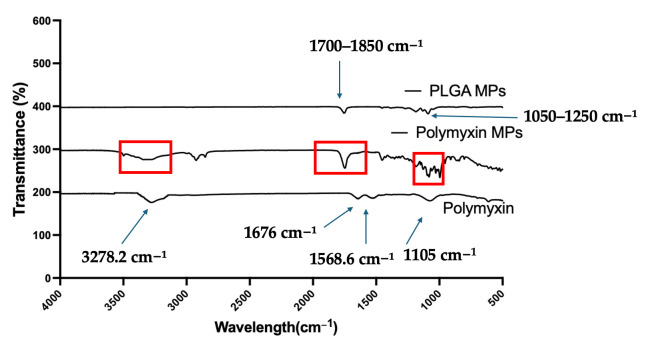
FTIR spectra in the range of 4000–500 cm^−1^ were recorded and analyzed for free Polymyxin B, blank PLGA microparticles, and Polymyxin B-loaded PLGA microparticles. The loaded formulation exhibited reduced intensity of the Polymyxin B amide (~1676 cm^−1^) and O–H/N–H (~3300 cm^−1^) bands, indicating hydrogen bonding and successful drug encapsulation within the PLGA matrix.

FTIR Spectroscopy: Polymyxin B revealed a characteristic peak at 3278.2 cm^−1^, respective to O–H stretching of the carboxylic group overlapping with N–H bending of the primary amine [34]. A distinct band at 1676 cm^−1^ was ascribed to C=O stretching of the primary amide. Furthermore, peaks at 1568.6 cm^−1^ and 1105 cm^−1^ were related to the C=C stretching of the aromatic ring from phenylalanine at position 6 and C–N stretching of the amine group, respectively.

(Figure 2) FTIR spectra of PLGA microparticles showed characteristic peaks at 1700–1850 cm^−1^ associated with the C=O stretching of the carbonyl group, and at 1050–1250 cm^−1^ indicating C–O stretching [35,36].

FTIR spectra of Polymyxin B loaded PLGA microparticles displayed characteristic PLGA peaks at ~1750 cm^−1^ and 1080–1180 cm^−1^. The amide (~1676 cm^−1^) band of Polymyxin B was present but with reduced intensity and partial overlap with PLGA bands, while the broad O–H/N–H stretching band (~3300 cm^−1^) was reduced, suggesting hydrogen bonding interactions. These spectral modifications confirm the successful encapsulation of Polymyxin B within the PLGA matrix.

### 3.2. In Vitro Release Profile of Polymyxin Microparticles

**Figure 3:**
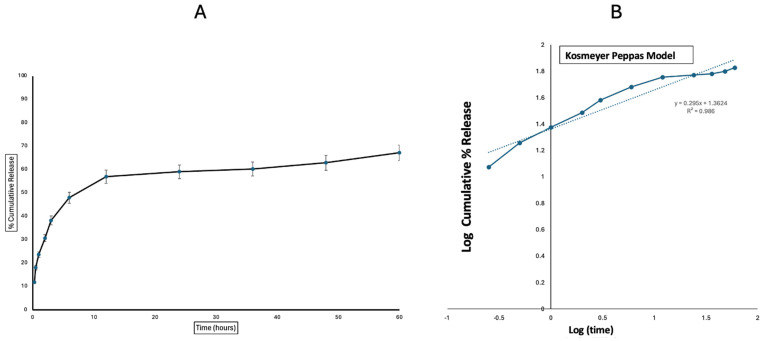
(**A**) In vitro release profile of Polymyxin B from PLGA microparticles over 60 h (n = 3), showing an initial burst (~24% at 1 h) followed by sustained release (~57% at 12 h) and plateauing at ~67% by 60 h. The release half-life was approximately 8 h, indicating controlled and prolonged drug release from the PLGA matrix. (**B**) The cumulative release was fitted to the Korsmeyer–Peppas kinetic model by plotting log cumulative % drug release vs. log time.

The in vitro release profile of Polymyxin B from Polymyxin B-loaded PLGA MPs was evaluated over 60 h. An initial burst release was observed within the first hour, with approximately 24% of Polymyxin B released at 1 h. This was followed by a sustained release phase, reaching around 48% at 6 h and 57% at 12 h. The half-life of release was ~8 h. After 24 h, the release gradually plateaued, with 63% of the drug released at 48 h and 67% at 60 h, indicating the controlled and prolonged release behavior of Polymyxin B from the microparticles. The release kinetics suggest that the PLGA matrix effectively modulates the drug release, which is beneficial for maintaining therapeutic levels over an extended period (Figure 3).

The Polymyxin B MPs were found to match the Korsmeyer–Peppas model based on the most suitable fit showing the strongest correlation value (R^2^), which was 0.986 (Table 3).

### 3.3. Assessment of the Cytotoxicity of Polymyxin B Microparticles

**Figure 4:**
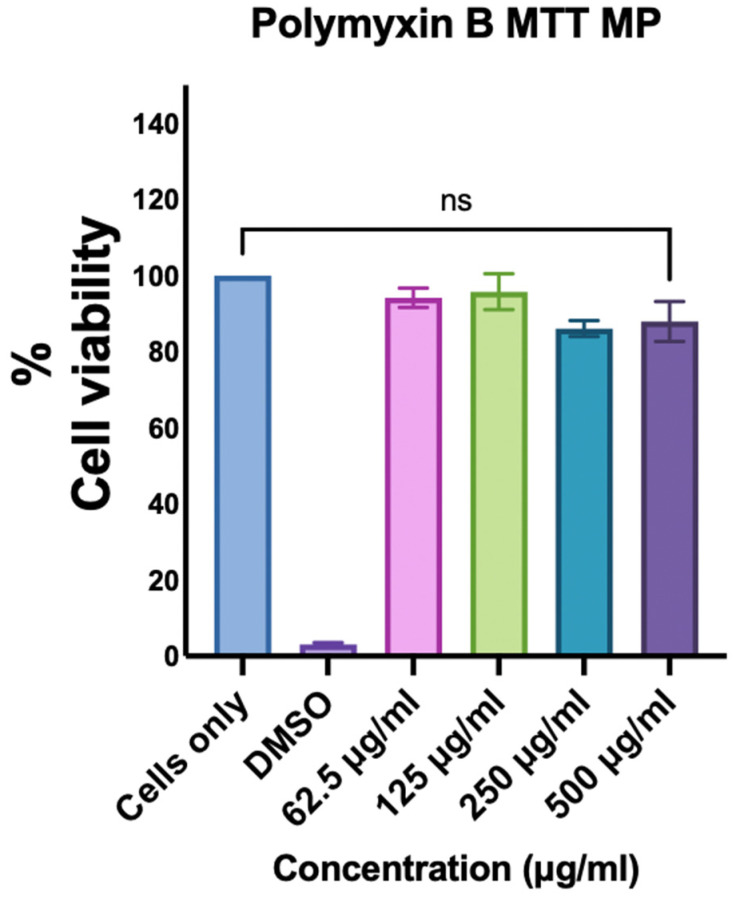
Dendritic cells are not cytotoxically affected by Polymyxin B microparticles. Briefly, DC 2.4 were cultured for 24 h after receiving to escalating doses of Polymyxin B MPs. MTT reagent, which uses the attenuating capacity of live cells to quantify cell viability, was used to analyze the cytotoxicity. The data are presented in triplicate with statistical analysis showing *p*-values > 0.05 as nonsignificant.

Dendritic cells were used to assess the Polymyxin B MPs’ cytotoxicity. The MTT test was used in this in vitro study. The cells were killed using the hazardous solvent DMSO as a positive control. Conversely, cell lines that had not been treated with Polymyxin B MPs served as negative controls. To assess cytotoxicity, the dendric cells were exposed to various doses of Polymyxin B MPs. From 500 μg/mL to lower concentrations, Polymyxin B MPs showed no significant cytotoxicity toward dendritic cells compared to cells only (Figure 4).

### 3.4. Qualitative and Quantitative Evaluation of Autophagosomes

**Figure 5:**
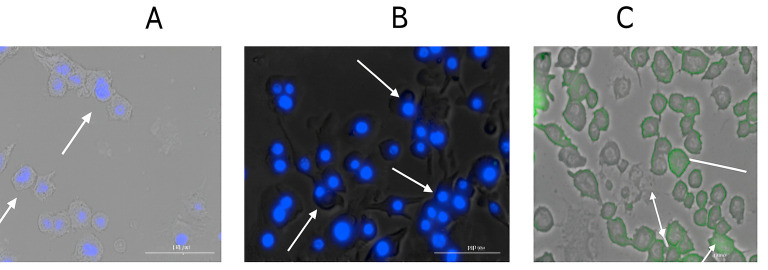
Evaluation of autophagy in dendritic cells treated with different groups. Imaging using fluorescent microscope (**A**). Cells only (negative control). (**B**). Polymyxin suspension. (**C**). Polymyxin MP. Qualitatively, Hoechst 33342 Nuclear stain was used to stain the cell nucleus, which appeared blue, and CYTO-ID^®^ dye was used to stain the autophagosomes, which appeared green as indicated by the arrows. Compared to the Polymyxin B suspension group, the Polymyxin B MP significantly increased the autophagy induced in dendritic cells.

**Figure 6:**
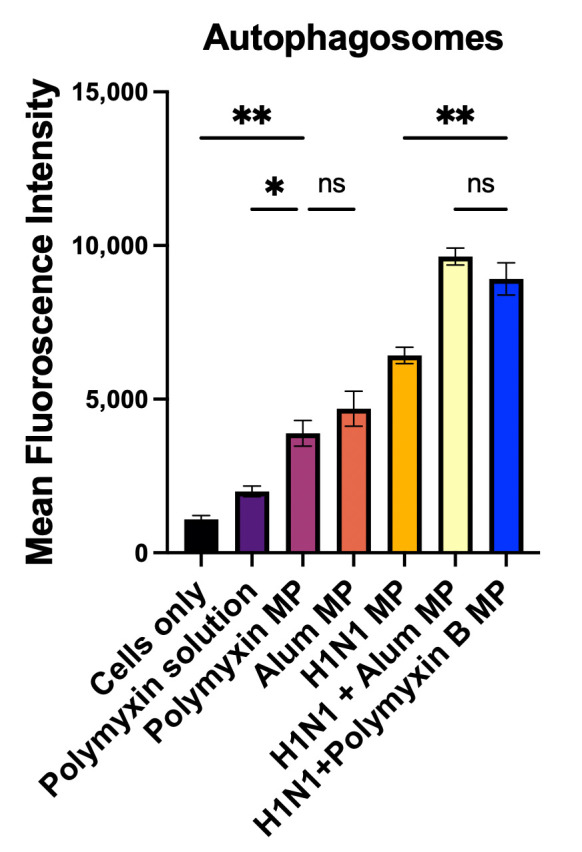
Flow cytometric assessment of autophagy was performed. Quantitative autophagosome formation was considerably higher in H1N1 + Polymyxin B MPs than in H1N1 MPs alone, but there was no significant difference between H1N1 + Polymyxin B MPs and H1N1 + Alum MPs. Data are presented in triplicate; statistical significance was determined using one-way ANOVA followed by Tukey’s post hoc multiple comparisons test. Statistical analysis showing *p* > 0.05 (nonsignificant), *p* ≤ 0.05 (*), *p* ≤ 0.01 (**). A *p*-value of less than 0.05 was deemed statistically significant in every experiment.

In this experiment, we evaluated the ability of Polymyxin B-loaded MPs to promote autophagy in dendritic cells. Representative images (Figure 5A–C) show increased autophagosome generation in the Polymyxin B MP group compared to untreated cells and the Polymyxin B suspension group. Quantitative analysis (Figure 6) confirmed a higher percentage of autophagy-positive cells following Polymyxin B MP treatment relative to the suspension form, suggesting that the microparticle carrier boosts autophagosome induction. Autophagosome generation in the Alum MP group was not significantly different from that observed with Polymyxin B MPs. Furthermore, dendritic cells treated with H1N1 + Polymyxin B MPs exhibited significantly greater autophagosome formation compared to H1N1 MPs alone, while no significant difference was noted between H1N1 + Polymyxin B MPs and H1N1 + Alum MPs.

### 3.5. In Vitro Immunogenicity Evaluation of Adjuvant Effect Using Griess Assay for Nitrite Detection

**Figure 7:**
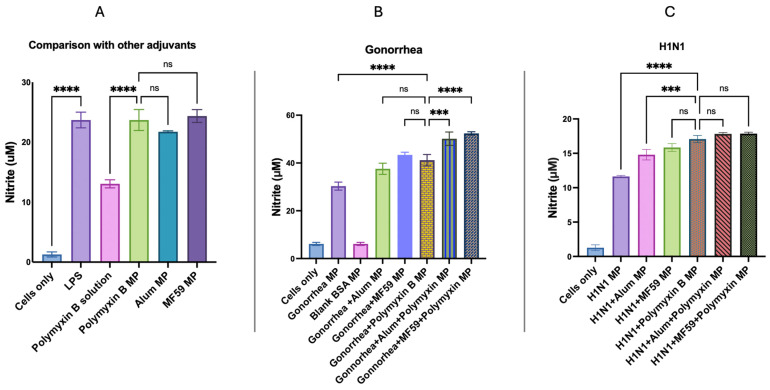
Nitric oxide production by dendritic cells as determined by Griess’ assay. (**A**) DC 2.4 were incubated with lipopolysaccharide (LPS), Polymyxin B, and FDA-licensed adjuvant (Alum and MF59) microparticles. (**B**) Nitric oxide release from dendritic cells incubated with blank bovine serum albumin (BSA) microparticles, gonorrhea vaccine with Polymyxin B, Alum, MF59^®^, or combinations of the adjuvants. (**C**) Nitric oxide release from dendritic cells incubated with H1N1 influenza vaccine with Polymyxin B, Alum, MF59, or combinations of the adjuvants. Results are presented in triplicate, with statistical analysis showing *p* > 0.05 (nonsignificant), *p* ≤ 0.001 (***), and *p* ≤ 0.0001 (****). A *p*-value of less than 0.05 was deemed statistically significant in every experiment.

In Figure 7A, it was observed that the dendritic cells released significantly higher amounts of nitric oxide when exposed to Polymyxin B MPs in comparison to Polymyxin B solution owing to the microparticulate nature. The nitric oxide released when exposed to Polymyxin MPs was not significantly different than established FDA-licensed adjuvant (Alum and MF59^®^ InvivoGen, San Diego, CA, USA) MPs.

In Figure 7B, the nitric oxide release from Polymyxin B MPs in combination with the gonorrhea vaccine was significantly higher than the gonorrhea vaccine only. In the combination of gonorrhea vaccine with Polymyxin B MPs, there was no significant difference in the release of nitric oxide from the dendritic cells upon exposure to the gonorrhea vaccine microparticles in combination with the conventional FDA-licensed adjuvant (MF59^®^ and Alum) MPs.

When Polymyxin B MPs were combined with Alum or MF59 in the gonorrhea vaccine, it markedly enhanced nitric oxide release compared to the gonorrhea vaccine with Polymyxin B alone. These results suggested that Polymyxin B MPs demonstrated an adjuvant effect with the gonorrhea vaccine.

In Figure 7C, the nitric oxide release from Polymyxin B microparticles in combination with the microparticulate H1N1 influenza vaccine was significantly higher than the microparticulate H1N1 vaccine only. Dendritic cells exposed to H1N1 vaccine microparticles in combination with Polymyxin B exhibited significantly higher nitric oxide compared to those treated with H1N1 MPs formulated with the traditional FDA-licensed adjuvant Alum and a comparable response to the MF59-adjuvanted H1N1 vaccine. When Polymyxin B MPs were combined with Alum or MF59 in the H1N1 vaccine, it showed no significant difference in nitric oxide release compared to the H1N1 vaccine with Polymyxin B alone. This suggested that microparticulate Polymyxin B showed an adjuvant effect with the microparticulate influenza vaccine but did not increase responses in combination with Aum/MF59 adjuvants.

**Figure 8:**
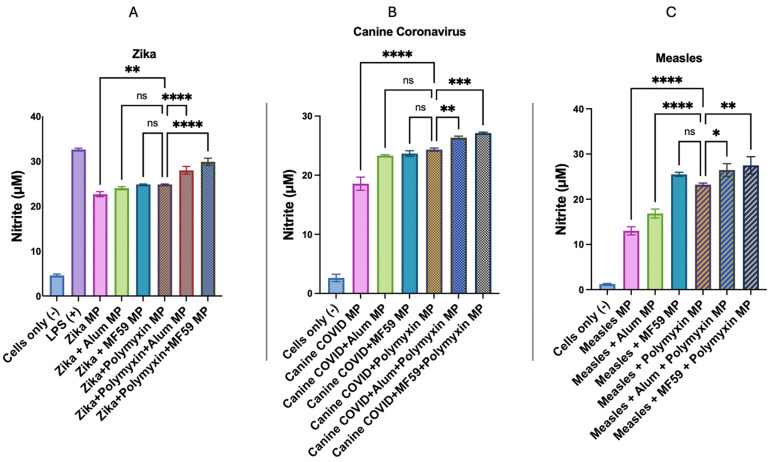
Nitric oxide production by dendritic cells as determined by Griess’ assay. Data are expressed in triplicates as mean ± standard error of the mean. (**A**) Nitric oxide release from dendritic cells incubated with lipopolysaccharide (LPS), Zika vaccine with Polymyxin B, Alum, MF59^®^, or combinations of the adjuvants. (**B**) Nitric oxide release from dendritic cells pulsed with canine coronavirus vaccine with Polymyxin B, Alum, MF59^®^, or combinations of the adjuvants. (**C**) Nitric oxide release from dendritic cells pulsed with measles vaccine with Polymyxin B, Alum, MF59^®^, or combinations of the adjuvants. Statistical analysis showing *p* > 0.05 (nonsignificant), *p* ≤ 0.05 (*), *p* ≤ 0.01 (**), *p* ≤ 0.001 (***), and *p* ≤ 0.0001 (****). A *p*-value of less than 0.05 was deemed statistically significant in every experiment.

From Figure 8A, it was observed that the dendritic cells released significantly higher levels of nitric oxide when incubated with Polymyxin B MPs in combination with the microparticulate Zika vaccine as compared to the microparticulate Zika vaccine only. No significant difference was observed in nitric oxide release from dendritic cells exposed to Zika vaccine MPs combined with FDA-licensed adjuvant microparticles (Alum or MF59^®^), compared with those exposed to Zika vaccine MPs along with Polymyxin B MPs. When Polymyxin B MPs were combined with Alum or MF59 MPs in the Zika vaccine, it markedly enhanced nitric oxide release compared to the Zika vaccine with Polymyxin B alone. These results show that microparticulate Polymyxin B exhibited an adjuvant effect with the microparticulate Zika vaccine.

In Figure 8B, the nitric oxide release from Polymyxin B MPs in combination with the microparticulate canine coronavirus vaccine was significantly higher than the microparticulate canine coronavirus vaccine only. Dendritic cells exposed to canine coronavirus vaccine MPs + Polymyxin B MPs exhibited a nonsignificant difference in nitric oxide response compared to those treated with canine coronavirus MPs + Alum MPs and canine coronavirus MPs + MF59 MPs. When Polymyxin B MPs were combined with Alum or MF59 in the canine coronavirus vaccine, it showed significant difference in nitric oxide release compared to the canine coronavirus vaccine with Polymyxin B alone. These results demonstrated that microparticulate Polymyxin B showed an adjuvant effect with the microparticulate canine coronavirus vaccine.

In Figure 8C, the nitric oxide release from Polymyxin B MPs in combination with the microparticulate measles vaccine was significantly higher than the microparticulate measles vaccine only. Dendritic cells exposed to measles MPs + Polymyxin B MPs exhibited a significantly higher nitric oxide response compared to those treated with measles MPs + Alum MPs and nonsignificant difference with measles MPs + MF59 MPs. When Polymyxin B MPs were combined with Alum or MF59 in the measles vaccine, it showed a significant difference in nitric oxide release compared to the measles vaccine with Polymyxin B alone. These results demonstrated that microparticulate Polymyxin B showed an adjuvant effect with the microparticulate measles vaccine.

### 3.6. Evaluation of Antigen-Presenting and Co-Stimulatory Molecule Expression by Flow Cytometry

**Figure 9:**
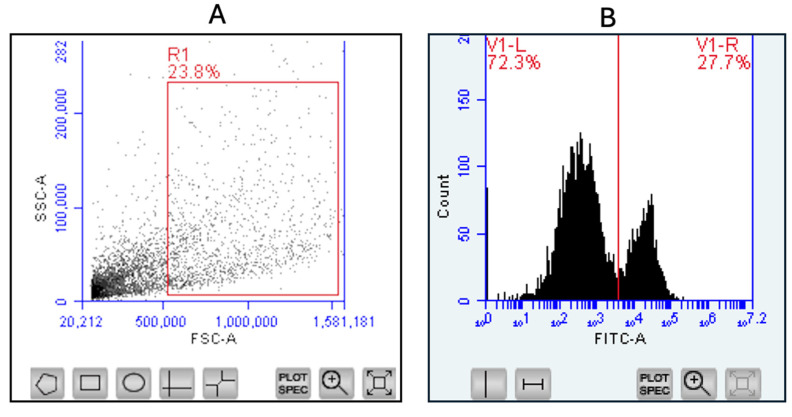
Gating strategy for all flow cytometry experiments. Panel (**A**) shows a representative dot plot of flow cytometry data used for initial gating, based on forward scatter (FSC-A) and side scatter (SSC-A), which select cells by size and granularity. The rectangular gate (R1) encloses 23.8% of events, identifying the main cell population and excluding debris. Panel (**B**) displays a histogram of fluorescence intensity (FITC-A), where a vertical gate differentiates between unstained (negative) and marker-positive (right) cells. Gate thresholds are set using unstained samples as controls, allowing accurate quantification of marker expression for all populations in the flow cytometry panel.

Panel A represents the initial gating strategy used in flow cytometry to select the population of interest based on forward scatter (FSC-A) and side scatter (SSC-A), which correspond to cell size and granularity, respectively. The gate, shown as a red rectangle labeled R1, includes 23.8% of the total events, allowing for the exclusion of debris and selection of viable cells. Panel B displays fluorescence intensity data for a specific marker (such as FITC-labeled antibody), where a vertical gate divides the histogram into two regions: V1-L (negative population, 72.3%) and V1-R (positive population, 27.7%). The gate position is set after analyzing the unstained control, ensuring accurate discrimination between unstained and marker-positive cells for all subsequent samples. This gating approach remains standard across all markers in the flow cytometry experiments (Figure 9).

**Figure 10:**
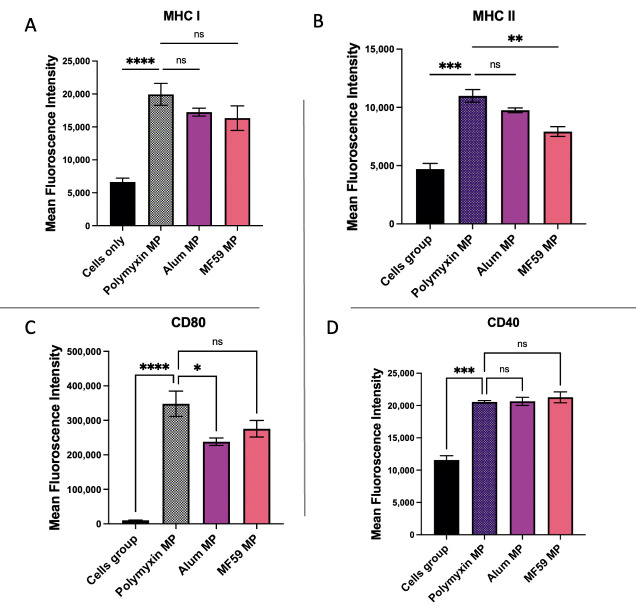
Murine dendritic cell surface expression of MHC I (**A**) and MHC II (**B**) together with CD80 (**C**) and CD40 (**D**). Cells were incubated with Polymyxin B MPs, Alum MPs, or MF59 MPs. Surface expression was analyzed using flow cytometry after staining with FITC-conjugated MHC I and MHC II and APC-conjugated CD40 and CD80. Data are presented in triplicate. Statistical analysis showing *p* > 0.05 (nonsignificant), *p* ≤ 0.05 (*), *p* ≤ 0.01 (**), *p* ≤ 0.001 (***), and *p* ≤ 0.0001 (****). A *p*-value of less than 0.05 was deemed statistically significant in every experiment.

To determine which molecules were upregulated by Polymyxin B, the surface expression of MHC I, MHC II, CD40, and CD80 on dendritic cells was evaluated using flow cytometry. As illustrated in Figure 10, for all markers examined, Polymyxin B MPs induced significantly higher expression compared to the untreated dendritic cells control group.

MHC I expression in dendritic cells treated with Polymyxin B MPs was significantly comparable to that observed with FDA-licensed adjuvant MPs (Alum and MF59). MHC II expression was significantly higher in the Polymyxin B MP group compared to MF59 MPs and was comparable to Alum MPs. CD80 expression on dendritic cells was significantly elevated in the Polymyxin B MP group compared to Alum MPs, while showing no significant difference relative to MF59 MPs. CD40 expression in the Polymyxin B MP group did not differ significantly from either Alum or MF59 MPs.

**Figure 11:**
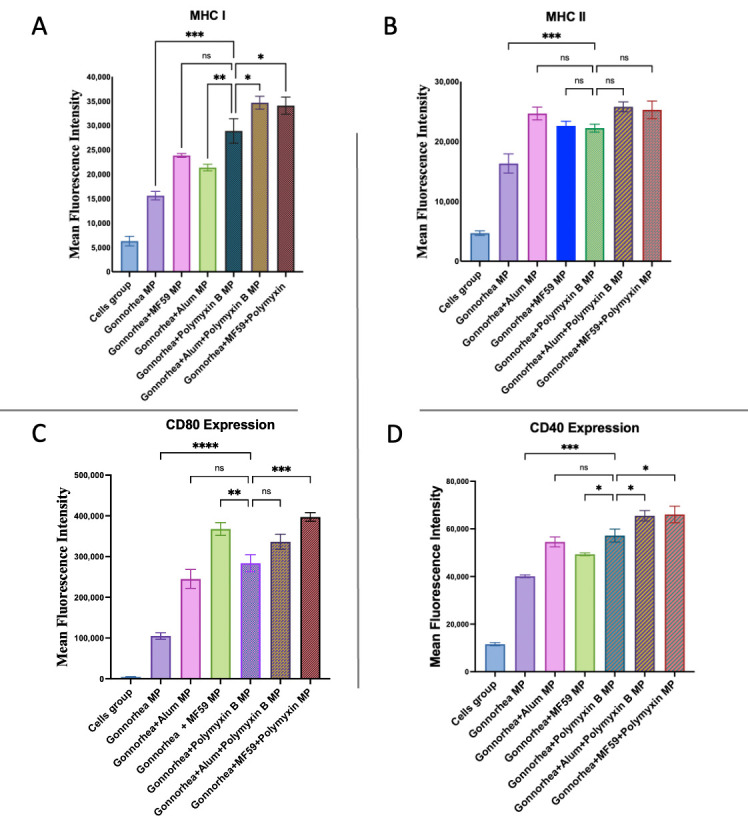
Murine dendritic cell surface expression of MHC I (**A**) and MHC II (**B**) together with CD80 (**C**) and CD40 (**D**). Cells were incubated with gonorrhea vaccine alone and in addition to Polymyxin B MPs, Alum MPs, or MF59 MPs. Surface expression was analyzed using flow cytometry after staining with FITC-conjugated MHC I and MHC II and APC-conjugated CD40 and CD80. Data are expressed in triplicate. Statistical analysis showing *p* > 0.05 (nonsignificant), *p* ≤ 0.05 (*), *p* ≤ 0.01 (**), *p* ≤ 0.001 (***), and *p* ≤ 0.0001 (****). A *p*-value of less than 0.05 was deemed statistically significant in every experiment.

To further assess the adjuvant effect of Polymyxin B MPs with bacterial vaccine candidates, we analyzed the expression of MHC I, MHC II, CD40, and CD80 on dendritic cells after exposure to microparticulate gonorrhea vaccine formulations (Figure 11). When combined with the gonorrhea MPs, Polymyxin B MPs significantly enhanced the expression of both antigen-presenting molecules and co-stimulatory molecules compared to vaccine MPs alone.

Cells treated with the gonorrhea vaccine plus Polymyxin B MPs showed significantly higher MHC I expression than those treated with the gonorrhea vaccine plus Alum MPs, and a comparable response to vaccine plus MF59 MPs. The addition of Alum or MF59 to Polymyxin B MPs further increased MHC I expression in comparison to the gonorrhea plus Polymyxin group. MHC II expression was significantly similar across groups treated with the gonorrhea vaccine plus Polymyxin B MPs, Alum MPs, or MF59 MPs. Adding Alum or MF59 to Polymyxin B MPs did not further enhance MHC II expression in comparison to the Polymyxin-adjuvanted gonorrhea vaccine. CD80 expression with Polymyxin B MPs was significantly lower than with MF59-adjuvanted gonorrhea vaccine but not significantly different from the Alum-adjuvanted gonorrhea vaccine. The incorporation of MF59 as a combination adjuvant with the Polymyxin B formulation significantly increased CD80 expression in comparison to the Polymyxin-adjuvanted gonorrhea vaccine, whereas Alum did not. CD40 expression was significantly higher in the gonorrhea vaccine group when adjuvanted with Polymyxin B MPs than with MF59 and not significantly different from Alum. Both Alum and MF59 significantly enhanced CD40 expression when used as a combination adjuvant with Polymyxin B MPs in comparison to the Polymyxin-adjuvanted gonorrhea vaccine.

**Figure 12:**
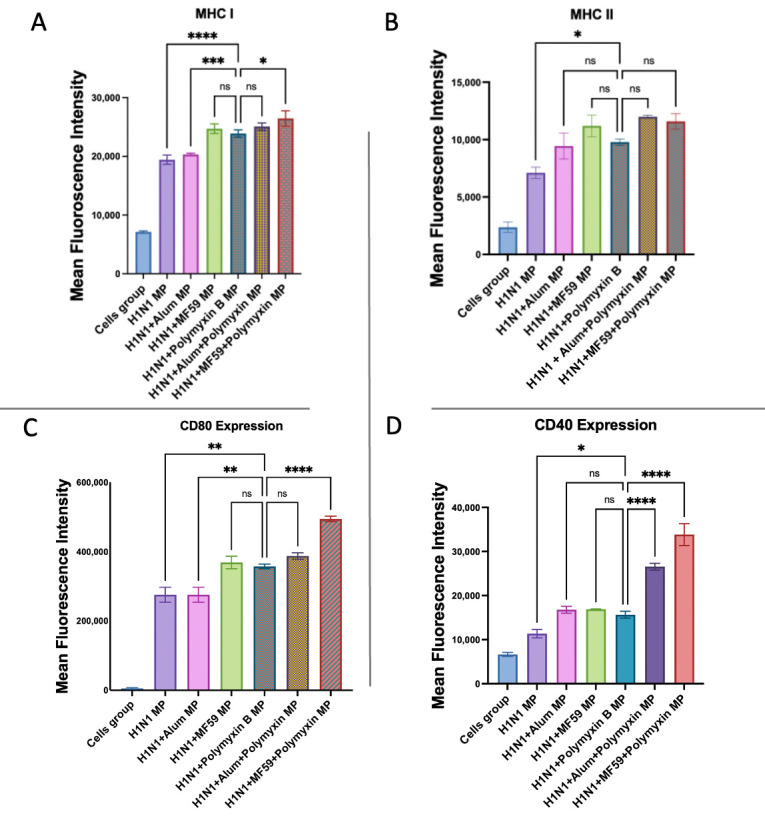
Murine dendritic cell surface expression of MHC I (**A**) and MHC II (**B**) together with CD80 (**C**) and CD40 (**D**). Cells were incubated with influenza H1N1 vaccine alone and in addition to Polymyxin B MPs, Alum MPs, or MF59 MPs. Surface expression was analyzed using flow cytometry after staining with FITC-conjugated MHC I and MHC II and APC-conjugated CD40 and CD80. Data are presented in triplicate, with statistical analysis showing *p* > 0.05 (nonsignificant), *p* ≤ 0.05 (*), *p* ≤ 0.01 (**), *p* ≤ 0.001 (***), and *p* ≤ 0.0001 (****). A *p*-value of less than 0.05 was deemed statistically significant in every experiment.

As shown in Figure 12, cells were treated with H1N1 vaccine MPs alone, or in combination with Alum MPs, MF59 MPs, Polymyxin B MPs, or Polymyxin B MPs plus Alum/MF59. When combined with the H1N1 vaccine, Polymyxin B MPs significantly increased the expression of both antigen-presenting molecules and co-stimulatory molecules compared to vaccine MPs alone.

Dendritic cells exposed to H1N1 + Polymyxin B MPs exhibited significantly higher MHC I expression than H1N1 + Alum MPs, and were comparable to H1N1 + MF59 MPs. The combination of MF59 and Polymyxin B MPs in the H1N1 influenza vaccine significantly increased MHC I expression in comparison to the Polymyxin-adjuvanted H1N1 vaccine, but the addition of Alum did not. There were no significant differences in the MHC II expression among H1N1 + Polymyxin B, H1N1 + Alum, or H1N1 + MF59 MPs. Adding Alum or MF59 to Polymyxin B MPs did not significantly increase MHC II expression further in comparison to the Polymyxin-adjuvanted H1N1 vaccine.

The CD80 expression in H1N1 + Polymyxin B MPs was significantly similar to H1N1 + MF59 MPs and higher than H1N1 + Alum MPs. The combination of Polymyxin B MPs with MF59 in the H1N1 influenza vaccine further significantly increased CD80 expression in comparison to Polymyxin H1N1 + Polymyxin B, whereas adding Alum did not. There were no significant differences in the CD40 expression among H1N1 + Polymyxin B, H1N1 + Alum, or H1N1 + MF59 MPs. Incorporating either Alum or MF59 with Polymyxin B MPs significantly increased CD40 expression relative to H1N1 + Polymyxin B MPs.

**Figure 13:**
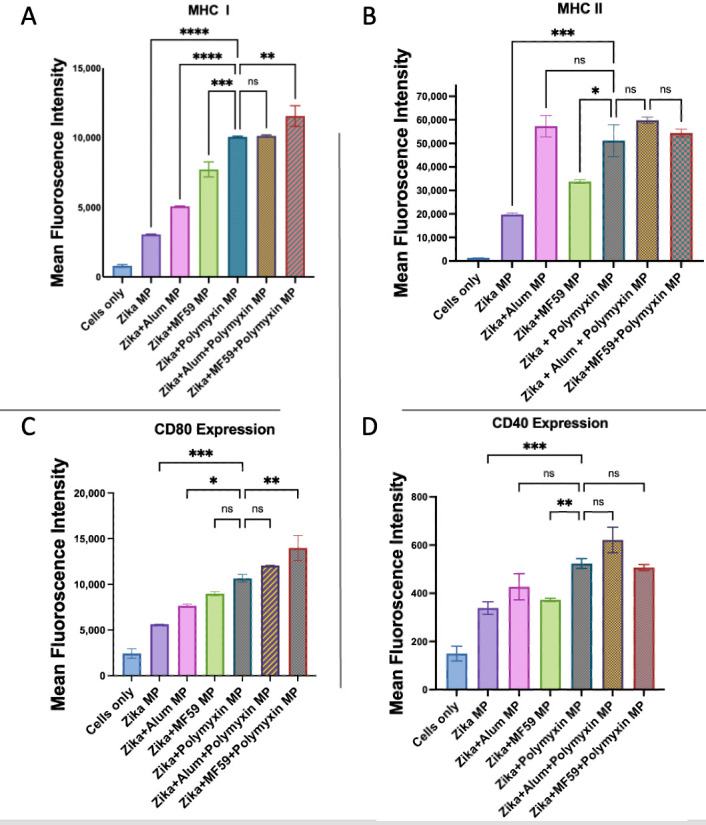
Murine dendritic cell surface expression of MHC I (**A**) and MHC II (**B**) together with CD80 (**C**) and CD40 (**D**). Cells were incubated with Zika vaccine alone and in addition to Polymyxin B MPs, Alum MPs, or MF59 MPs. Surface expression was analyzed using flow cytometry following staining with FITC-conjugated MHC I and MHC II and APC-conjugated CD40 and CD80. Data are presented in triplicate, with statistical analysis showing *p* > 0.05 (nonsignificant), *p* ≤ 0.05 (*), *p* ≤ 0.01 (**), *p* ≤ 0.001 (***), and *p* ≤ 0.0001 (****). A *p*-value of less than 0.05 was deemed statistically significant in every experiment.

As shown in Figure 13, when combined with the Zika vaccine, Polymyxin B MPs significantly enhanced the expression of both antigen-presenting and co-stimulatory molecules compared to vaccine MPs alone. Dendritic cells exposed to Zika + Polymyxin B MPs exhibited significantly higher MHC I expression than those treated with Zika + Alum MPs or Zika + MF59 MPs. The addition of MF59 to Polymyxin B MPs in the Zika vaccine significantly increased the MHC I expression in comparison to the Polymyxin-adjuvanted Zika vaccine, whereas Alum did not. The MHC II expression was significantly higher in Zika + Polymyxin B MPs compared to Zika + MF59 MPs and was not significantly different from Zika + Alum MPs. The addition of MF59 or Alum to Polymyxin B MPs in the Zika vaccine did not significantly boost the MHC II expression in comparison to the Polymyxin-adjuvanted Zika vaccine. The CD80 expression was significantly higher in Zika + Polymyxin B MPs than in Zika + Alum MPs and comparable to Zika + MF59 MPs. Interestingly, adding MF59 to Polymyxin B MPs significantly increased CD80 expression in comparison to the Polymyxin-adjuvanted Zika vaccine, whereas Alum did not. The CD40 expression in Zika + Polymyxin B MPs was significantly similar to Zika + Alum MPs and higher than Zika + MF59 MPs. Using Polymyxin B MPs as a combination adjuvant with either MF59 or Alum in the Zika vaccine did not further significantly increase CD40 expression in comparison to the Polymyxin-adjuvanted Zika vaccine.

**Figure 14:**
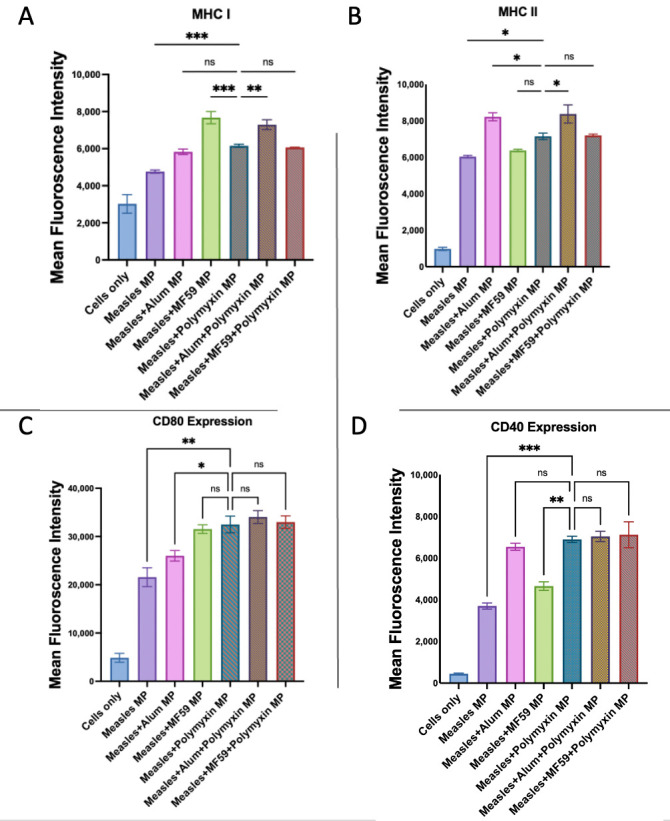
Murine dendritic cell surface expression of MHC I (**A**) and MHC II (**B**) together with CD80 (**C**) and CD40 (**D**). Cells were incubated with measles vaccine alone and in addition to Polymyxin B MPs, Alum MPs, or MF59 MPs. Surface expression was analyzed using flow cytometry following staining with FITC-conjugated MHC I and MHC II and APC-conjugated CD40 and CD80. Data are presented in triplicate, with statistical analysis showing *p* > 0.05 (nonsignificant), *p* ≤ 0.05 (*), *p* ≤ 0.01 (**) and *p* ≤ 0.001 (***). A *p*-value of less than 0.05 was deemed statistically significant in every experiment.

In Figure 14, when combined with the measles vaccine, Polymyxin B MPs significantly enhanced the expression of antigen-presenting and co-stimulatory molecules compared to measles MPs alone. Measles + Polymyxin B MPs induced significantly lower MHC I expression than measles + MF59 MPs and showed no significant difference from measles + Alum MPs. Adding Alum to Polymyxin B MPs in the measles vaccine significantly increased MHC I expression in comparison to the Polymyxin-adjuvanted measles vaccine, while MF59 had no significant effect. Measles + Polymyxin B MPs induced lower MHC II expression than measles + Alum MPs and were not significantly different from measles + MF59 MPs. The addition of Alum, but not MF59, in the Polymyxin B-adjuvanted measles vaccine significantly increased the expression in comparison to the Polymyxin-adjuvanted measles vaccine.

The CD80 expression was significantly higher with measles + Polymyxin B MPs than with measles + MF59 MPs and comparable to measles + Alum MPs. Neither MF59 nor Alum significantly increased expression when used as a combination adjuvant with Polymyxin B MPs in the measles vaccine in comparison to the Polymyxin-adjuvanted measles vaccine. The CD40 expression in measles + Polymyxin B MPs was significantly comparable to measles + Alum MPs and higher than measles + MF59 MPs. The co-adjuvant strategy with MF59 or Alum in the Polymyxin B-adjuvanted measles vaccine did not significantly increase CD40 expression in comparison to measles + Polymyxin B MPs.

**Figure 15:**
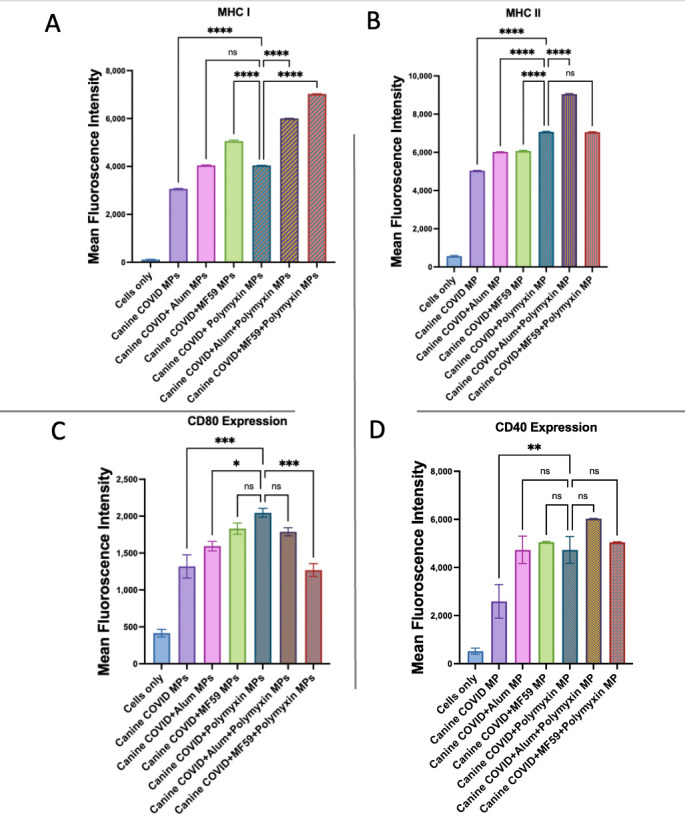
Murine dendritic cell surface expression of MHC I (**A**) and MHC II (**B**) together with CD80 (**C**) and CD40 (**D**). Cells were incubated with canine coronavirus vaccine alone and in addition to Polymyxin B MPs, Alum MPs, or MF59 MPs. Surface expression was analyzed using flow cytometry (n = 3) following staining with FITC-conjugated MHC I and MHC II and APC-conjugated CD40 and CD80. Statistical analysis showing *p* > 0.05 (nonsignificant), *p* ≤ 0.05 (*), *p* ≤ 0.01 (**), *p* ≤ 0.001 (***), and *p* ≤ 0.0001 (****). A *p*-value of less than 0.05 was deemed statistically significant in every experiment.

As shown in Figure 15, when combined with the canine coronavirus vaccine, Polymyxin B MPs significantly enhanced the expression of antigen-presenting and co-stimulatory molecules compared to vaccine MPs alone. Canine coronavirus + Polymyxin B MPs induced significantly lower MHC I expression than canine coronavirus + MF59 MPs and showed no significant difference from canine coronavirus + Alum MPs. Adding MF59 or Alum to Polymyxin B MPs in the canine coronavirus vaccine further significantly increased the MHC I expression in comparison to the Polymyxin-adjuvanted canine coronavirus vaccine. Canine coronavirus + Polymyxin B MPs induced higher MHC II expression than canine coronavirus + Alum MPs and canine coronavirus + MF59 MPs. The addition of Alum, but not MF59, to the Polymyxin B-adjuvanted canine coronavirus vaccine significantly increased MHC II expression in comparison to the Polymyxin-adjuvanted canine coronavirus vaccine.

The CD80 expression was significantly higher with canine coronavirus + Polymyxin B MPs than with canine coronavirus + Alum MPs and comparable to canine coronavirus + MF59 MPs. Adding MF59 significantly decreased expression when combined with Polymyxin B MPs in the coronavirus vaccine and adding Alum had no significant effect in comparison with canine coronavirus + Polymyxin B MPs. The CD40 expression in canine coronavirus + Polymyxin B MPs was significantly similar to canine coronavirus + Alum MPs and canine coronavirus + MF59 MPs. Combining MF59 or Alum with Polymyxin B MPs in the coronavirus vaccine did not significantly increase CD40 expression in comparison with canine coronavirus + Polymyxin B MPs.

## 4. Discussion

There is a great need for safer and more effective novel adjuvants incorporated into vaccines due to the quick spread of newly discovered infections. Pain, redness, and swelling at the local injection site are among the occasionally serious adverse effects of the existing adjuvants on the market or in clinical trials, along with tiredness, fever, headaches etc. A rise in anti-vaccination attitudes and the ensuing public health issues from under-vaccination have been caused by growing public fears about these negative effects. Given this, there is a growing need to develop new adjuvants to reduce systemic and local side effects while preserving vaccine effectiveness [37,38,39,40,41,42,43].

For the first time, we investigated the possibility of using Polymyxin B’s microparticulate formulation as a vaccine adjuvant. Polymyxin B was encapsulated using PLGA, a biodegradable, FDA-approved polymer that is biocompatible, provides controlled release drug delivery, and has been extensively studied as a carrier for medications, proteins, and various large biomolecules [32,33,34]. It has been discovered that circulating APCs can efficiently identify and consume MPs that range in size from 1000 nm to 3000 nm [36]. We have previously demonstrated encapsulation efficiencies for vaccine-loaded PLGA microparticles prepared using a double-emulsion solvent evaporation method, including 92% for H1N1 microparticles [44], 91.7% for iCCoV antigen microparticles [45], and 55–70% for Zika microparticles [46]. The size of Polymyxin B MPs was 2017 nm. The high positive or negative charges of these MPs help prevent the particles from clumping together when in suspension, maintaining the stability of colloidal suspensions [47]. The vaccine microparticles have a negative or positive charge depending on what they are encapsulated with [48]. The Polymyxin B MPs had a charge of −22.6 mV, indicating that there was no particle clumping from a high charge. The homogeneous size distribution of the particles is indicated by the low PDI of 0.296. Morphologically, the Polymyxin B MPs were spherical (Figure 1). Improved APC antigen uptake is indicated by a spherical shape [49].

The incorporation of Polymyxin B into the PLGA matrix led to the disappearance of several characteristic peaks in the FTIR spectrum of Polymyxin B MPs. These spectral modifications suggest that Polymyxin B is primarily encapsulated within the PLGA matrix, with minimal presence on the microparticle surface. The suppression of Polymyxin B’s stretching and bending vibrations, likely arising from hydrogen bonding or hydrophobic interactions with the PLGA, explains the absence of most of its characteristic peaks in the formulation. Together, these findings confirm the successful encapsulation of Polymyxin B within the PLGA matrix (Figure 2). Further, nearly 50% release of Polymyxin B by the polymer was observed close to 6 h (Figure 3), and various release models were assessed for the in vitro release kinetics investigations. For zero-order kinetics, this includes the percentage of cumulative Polymyxin B release versus time; for Korsmeyer–Peppas, it is the log percentage of cumulative Polymyxin B release versus log time; for Higuchi, it is the percentage of cumulative Polymyxin B release versus square root of time; and for first-order kinetics, it is the log percentage of cumulative Polymyxin B remaining versus time [50]. Table 3’s best fit with the highest correlation value (R^2^), 0.986, led us to the conclusion that the Polymyxin B encapsulated in nanoparticles fit the Korsmeyer–Peppas model. Furthermore, the magnitude of the release exponent (n) indicated that non-Fickian diffusion is the release mechanism. A complex release profile is produced by this release mechanism, which combines diffusion through the matrix with polymer-controlled processes including swelling, erosion, or relaxing [25].

Every candidate being studied as a possible vaccination adjuvant needs to first have its toxicity assessed in accordance with World Health Organization (WHO) recommendations [51]. We examined the toxicity profile of microparticulate Polymyxin B MPs to dendritic cells, a subset of APCs that initially come into contact with the microparticles when they are given in vivo. Even at concentrations of 500 μg/mL, we discovered that the Polymyxin B MPs microparticulate formulation was non-cytotoxic to dendritic cells. Our selected MTT concentrations (62.5–500 µg/mL) were chosen to reflect and bracket the exposure levels reported in previous in vivo studies using a 500 µg PMB dose in 6-week-old mice (18–21 g). Intranasal immunization with whole inactivated influenza vaccine combined with PMB induced robust influenza virus-specific IgA titer and conferred protection against lethal dose virus challenge compared with that observed in the immunized mice. Therefore, the upper end of our in vitro range (250–500 µg/mL) was intentionally included to mimic the transient high local concentrations that immune and epithelial cells may encounter shortly after dosing [52].

Further, dendritic cells rapidly took up Polymyxin B microparticles, which then aggregated in the vacuolar and cytosolic compartments. Numerous physiological and pathological mechanisms that control immunological responses, autoimmunity, cancer, infection, and neurological disorders have been linked to autophagy, a homeostatic process [53]. By removing invasive pathogens and promoting antigen presentation, autophagy enhances immunity and plays a critical part in host defense [54]. For MHCI and MHCII, autophagy entails antigen processing and presentation in macrophages or dendritic cells. We demonstrated increased autophagic vacuole formation in APCs both qualitatively and quantitatively. Qualitatively, exposure to Polymyxin B MPs led to enhanced autophagy induction compared to Polymyxin B solution due to the foreign microparticulate nature, as evidenced by green staining (Figure 5) [55]. Quantitatively, autophagosome formation in Polymyxin B MPs was compared with Alum MPs using H1N1 influenza as a model antigen. The results showed no significant difference between Alum and Polymyxin B MPs, and H1N1 + Polymyxin B MPs displayed autophagosome levels comparable to those observed with H1N1 + Alum MPs. Future work will include time-course-sensitive analysis to distinguish early versus sustained autophagy induction.

APC releases nitric oxide, a crucial innate immunogenicity marker, in response to antigen stimulation, as determined by the Griess assay [56,57]. Polymyxin B could generate nitrite in higher or similar amounts to the FDA-approved adjuvants (Figure 6 and Figure 7). The presentation of antigen is an essential criterion for promoting T cell responses in vaccines, as antigens are loaded onto the MHC molecules. Cytotoxic CD8+ T cells recognize antigens presented by MHC class I molecules, whereas helper T cells identify those presented by MHC class II molecules [58,59,60,61]. As a result, formulations of microparticulate vaccines have been thoroughly investigated and have been demonstrated to increase antigen immunogenicity. The APCs’ release of nitric oxide and the efficient activation of MHC I- and MHC II-mediated presentation and expression of their co-stimulatory molecules, CD80 and CD40, on the APC surface demonstrated that these microparticles were immunogenic. Autophagy activation and nitric oxide release can occur simultaneously, and their combined upregulation often marks adjuvant efficacy, with both mechanisms contributing to adjuvant efficacy but via partially distinct immunological pathways. While autophagy mainly enhances MHC-dependent antigen presentation and adaptive immunity, NO release boosts early innate responses and APC maturation [62].

Figure 8, Figure 9, Figure 10, Figure 11, Figure 12 and Figure 13 show that Polymyxin B MPs consistently enhanced dendritic cell activation across multiple vaccine platforms, upregulating MHC I, CD80, MHC II, and CD40, which presents to both CD4+ and CD8+ T cells. Compared to Alum, which generally lagged in MHC I and CD80 due to its Th2 bias, Polymyxin B was consistently superior, while its efficacy was largely comparable to MF59. When combined with MF59 or Alum, Polymyxin B sometimes further increased marker expression, while in other cases, no additional effect was observed, indicating that Polymyxin B is a potent adjuvant capable of functioning alone or in combination in only some cases to promote robust cellular and humoral immunity.

Limitations of the study include the lack of investigation of distinct cytokines, which would help in validating the adjuvant potential of Polymyxin B MPs as well as the potential mechanisms of Polymyxin B as an adjuvant, which should be explored in greater depth. We acknowledge that the absence of certified endotoxin-free antigen preparations is a limitation of the current study, and therefore residual endotoxin contamination cannot be fully excluded as a contributor to the observed nitric oxide levels. Future studies will involve testing Polymyxin B with more vaccine candidates and conducting in vivo studies to assess memory markers.

## 5. Conclusions

Polymyxin B is immunogenic and exhibits significant adjuvant activity with multiple vaccine candidates, including viral and bacterial antigens. In vitro studies and immune profiling studies indicate that Polymyxin B MPs are immunostimulatory, upregulating the MHC I and MHC II pathway, and are comparable to FDA-licensed adjuvants such as Alum and MF59. Our study results highlight the potential of Polymyxin B MPs as a versatile adjuvant platform for diverse vaccines.

## Figures and Tables

**Table 1:** A summary of the in vitro experiment’s treatment groups and doses.

Treatment Groups	Dose of Microparticles/Well
Alum MPs	100 µg/well
MF59 MPs	100 µg/well
Polymyxin MPs	100 µg/well
Gonorrhea MPs	100 µg/well
Gonorrhea MP + Alum MPs	100 µg/well + 50 µg/well
Gonorrhea MP + MF59 MPs	100 µg/well + 50 µg/well
Gonorrhea MP + Polymyxin B MPs	100 µg/well + 50 µg/well
Gonorrhea MP + Alum + Polymyxin B MPs	100 µg/well + 25 µg/well + 25 µg/well
Gonorrhea MP + MF59 + Polymyxin B MPs	100 µg/well + 25 µg/well + 25 µg/well
H1N1 MPs	100 µg/well
H1N1 MPs + Alum MPs	100 µg/well + 50 µg/well
H1N1 MPs + MF59 MPs	100 µg/well + 50 µg/well
H1N1 MPs + Polymyxin B MPs	100 µg/well + 50 µg/well
H1N1 MPs + Alum + Polymyxin B MPs	100 µg/well + 25 µg/well + 25 µg/well
H1N1 MPs + MF59 + Polymyxin B MPs	100 µg/well + 25 µg/well + 25 µg/well
Zika MPs	100 µg/well
Zika MPs + Alum MPs	100 µg/well + 50 µg/well
Zika MPs + MF59 MPs	100 µg/well + 50 µg/well
Zika MPs + Polymyxin B MPs	100 µg/well + 50 µg/well
Zika MPs + Alum + Polymyxin B MPs	100 µg/well + 25 µg/well + 25 µg/well
Zika MPs + MF59 + Polymyxin B MPs	100 µg/well + 25 µg/well + 25 µg/well
Measles MPs	100 µg/well
Measles MPs + Alum MPs	100 µg/well + 50 µg/well
Measles MPs + MF59 MPs	100 µg/well + 50 µg/well
Measles MPs + Polymyxin B MPs	100 µg/well + 50 µg/well
Measles MPs + Alum + Polymyxin B MPs	100 µg/well + 25 µg/well + 25 µg/well
Measles MPs + MF59 + Polymyxin B MPs	100 µg/well + 25 µg/well + 25 µg/well
Canine Coronavirus MPs	100 µg/well
Canine Coronavirus MPs + Alum MPs	100 µg/well + 50 µg/well
Canine Coronavirus MPs + MF59 MPs	100 µg/well + 50 µg/well
Canine Coronavirus MPs + Polymyxin B MPs	100 µg/well + 50 µg/well
Canine Coronavirus MPs + Alum + Polymyxin B MPs	100 µg/well + 25 µg/well + 25 µg/well
Canine Coronavirus MPs + MF59 + Polymyxin B MPs	100 µg/well + 25 µg/well + 25 µg/well

**Table 2:** Characterization of Polymyxin B MPs in respect to particle size (micrometers), recovery yield (%), polydispersity index, and zeta potential (n = 3).

Parameter	Polymyxin MPs
Size	2017 ± 300.7 nm
% Recovery yield	78% ± 5%
Polydispersity index	0.296 ± 0.0014
Zeta potential	−22.6 ± 4.1 mV

**Table 3:** Analysis of the R-square values of release kinetics of Polymyxin B from the PLGA MPs.

Model	R^2^
Zero-order	0.671
First-order	0.13
Higuchi	0.423
Korsmeyer–Peppas	0.986

## Data Availability

Dataset available on request from the authors.
